# Serum C-reactive protein and thioredoxin levels in subjects with mildly reduced glomerular filtration rate

**DOI:** 10.1186/1471-2369-11-7

**Published:** 2010-04-27

**Authors:** Shoko Tsuchikura, Tetsuo Shoji, Naoko Shimomura, Ryusuke Kakiya, Masanori Emoto, Hidenori Koyama, Eiji Ishimura, Masaaki Inaba, Yoshiki Nishizawa

**Affiliations:** 1 Department of Metabolism, Endocrinology and Molecular Medicine, Osaka City University Graduate School of Medicine, Osaka, Japan; 2 Department of Nephrology, Osaka City University Graduate School of Medicine, Osaka, Japan; 3 Division of Internal Medicine, Inoue Hospital, Suita, Japan

## Abstract

**Background:**

Chronic kidney disease (CKD) is a newly recognized high-risk condition for cardiovascular disease (CVD), and previous studies reported the changes in inflammation and oxidative stress in advanced stages of CKD. We compared the levels of serum biomarkers for inflammation and oxidative stress between subjects with normal and mildly reduced glomerular filtration rate (GFR).

**Methods:**

The subjects were 182 participants of a health check-up program including those with normal (≥ 90 mL/min/1.73 m^2^, N = 79) and mildly reduced eGFR (60-89 mL/min/1.73 m^2^, N = 103) which was calculated based on serum creatinine, age and sex. We excluded those with reduced eGFR < 60 mL/min/1.73 m^2^. No one had proteinuria. We measured serum levels of C-reactive protein (CRP) and thioredoxin (TRX) as the markers of inflammation and oxidative stress, respectively.

**Results:**

As compared with subjects with normal eGFR, those with mildly reduced eGFR had increased levels of both CRP and TRX. Also, eGFR was inversely correlated with these biomarkers. The associations of eGFR with these biomarkers remained significant after adjustment for age and sex. When adjustment was done for eight possible confounders, CRP showed significant association with systolic blood pressure, high density lipoprotein cholesterol (HDL-C) and non-HDL-C, whereas TRX was associated with sex significantly, and with eGFR and systolic blood pressure at borderline significance.

**Conclusions:**

We showed the increased levels of CRP and TRX in subjects with mildly reduced eGFR. The eGFR-CRP link and the eGFR-TRX link appeared to be mediated, at least partly, by the alterations in blood pressure and plasma lipids in these subjects.

## Background

Chronic kidney disease (CKD) is a newly recognized high-risk population for cardiovascular disease (CVD) [1]. The relative risk of death from myocardial infarction is 10-30 times higher in hemodialysis patients (CKD stage 5D) as compared to the general population [2]. Atherosclerotic vascular changes are present in patients with CKD not yet treated with hemodialysis [3-5] as well as in hemodialysis patients [6]. The risk for CVD increases in a stepwise manner as glomerular filtration rate (GFR) declines [7]. The increased risk of CVD in reduced GFR may be explained at least partly by impairment of classical risk factors including hypertension [8], dyslipidemia [9], and glucose intolerance/insulin resistance [10]. In addition, inflammation and increased oxidative stress [11,12] presumably contribute to the CKD-related excess risk for CVD [1].

Oxidative stress is determined by the balance between the production and elimination of reactive oxygen species (ROS) [13]. Since superoxide anion and other ROS are difficult to be evaluated reliably in clinical conditions due to their very short half-lives, more stable markers have been measured in biological specimens. For example, oxidative modifications of lipids, proteins, and nucleic acids can be evaluated by thiobarbituric acids-reactive substances (TBARS)[14,15], advanced oxidation protein products (AOPP), and 8-hydroxydeoxyguanodine (8-OHdG)[16], respectively. In addition, proteins that are secreted into the circulation in response to oxidative stress may serve as the biomarkers for oxidative stress. Thioredoxin (TRX) is among such proteins. TRX is a 12 kD protein, secreted by most cell types, with a redox-active dithiol/disulfide in the active site consensus sequence: -Cys-Gly-Pro-Cys-[17], showing anti-oxidative properties. Plasma TRX levels are increased in response to oxidative stress as shown in experimental [18] and human studies [19-21]. Also, serum TRX is known to be elevated in patients with increased oxidative stress, such as pancreatic cancer [22], hepatitis C virus infection [23], severe burn injury [24], acquired immunodeficiency syndrome (AIDS) [25], rheumatoid arthritis [26], heart failure [27], steato hepatitis [28], and interstitial lung disease [29].

Uremia is considered as pro-oxidant state[11,30]. Previous studies demonstrated the elevated levels of biomarkers for oxidative modification of lipids[31] and proteins[15] in dialysis patients, and in advanced stages of CKD prior to renal replacement therapy[32]. So far, however, information is limited regarding possible changes in inflammation and oxidative stress among subjects with mild reduction of renal function[33-35].

In the present study, we measured C-reactive protein (CRP) and TRX as biomarkers for inflammation and oxidative stress, and compared them between subjects with normal and mildly reduced glomerular filtration rate (GFR).

## Methods

### Subjects

**Figure 1:**
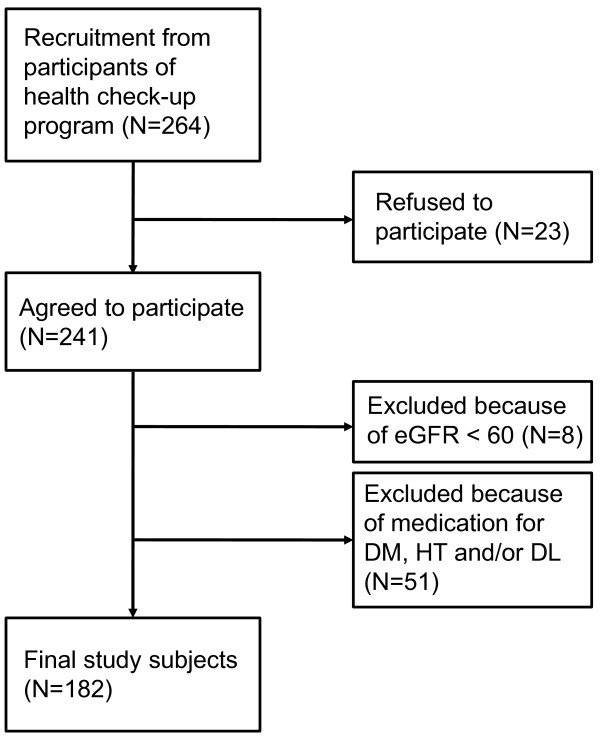
**Recruitment and selection of the subjects**. Abbreviations: DM, diabetes mellitus; HT, hypertension; DL, dyslipidemia.

The subjects were recruited from 264 consecutive participants of a health check-up program at the Osaka Health Promotion Center, Osaka, Japan. Twenty-three individuals refused to participate, and 241 subjects gave written informed consent to take part in the study. From the 241 people, we excluded 8 subjects with reduced eGFR < 60 mL/min/1.73 m^2 ^and 51 subjects taking medications for diabetes mellitus, hypertension, and/or dyslipidemia, to avoid possible influence of these medications to oxidative stress biomarkers. The remaining 182 individuals were the final subjects of this study (Figure 1). Table 1 summarizes the characteristics of the final subjects. No one had proteinuria by a dip-stick method. According to the criteria by the Kidney Disease Improving Global Outcomes (KDIGO)[36], 79 subjects had normal eGFR (90 ml/min/1.73 m^2 ^or higher), and 103 subjects showed mildly reduced eGFR (60-89 ml/min/1.73 m^2^). This study was carried out in compliance with the Helsinki Declaration, and approved by the ethics committee at Osaka City University Graduate School of Medical School.

**Table 1:** Characteristics of the subjects and summary of the measurements

	Median (25th-75th percentile)
Age	53 (39--63)
Male sex (%)	38*
BMI (kg/m^2^)	22.3 (20.1--24.1)
Systolic BP (mmHg)	121 (108--133)
Diastolic BP (mmHg)	70 (63--76)
Non-HDL-cholesterol (mg/dl)	144 (118--168)
HDL-cholesterol (mg/dl)	60 (49--73)
Smokers (%)	38*
Fasting plasma glucose (mg/dl)	97 (92--102)
eGFR (ml/min/1.73 m^2^)	85 (76--96)
CRP (mg/dl)	0.04 (0.02--0.09)
TRX (ng/ml)	10.2 (7.9--12.4)

Values are medians (25th-75th percentile) for continuous variables, and percentage* for sex and smokers.

Abbreviations are: BMI, body mass index; BP, blood pressure; HOMA-IR, insulin resistance index by homeostasis model assessment; HDL, high density lipoprotein; Non-HDL; non high density lipoprotein; eGFR; estimated glomerular filtration rate; CRP, C-reactive protein; TRX, thioredoxin.

### Estimation of glomerular filtration rate

Glomerular filtration rate (GFR) was estimated by the following equation:

where Cr is serum creatinine level by an enzymatic method. This equation was validated against the gold standard inulin clearance methods among Japanese individuals [37].

### Blood collection and measurements

Venous blood was collected after overnight fast into plastic tubes. After clotting at room temperature for 10 minutes, the tubes were chilled in ice, and serum was separated by centrifugation for 20 minutes at 4°C. Serum levels of TRX were measured within 3 days after sampling using frozen sera at -30°C. Other assays were performed immediately. CRP was assayed by a sensitive Latex-immunoassay (Denka Seiken, Tokyo) with a detection limit of 0.01 mg/dL. Serum TRX was quantified using a commercial ELISA kit for human TRX (Redox Bioscience Inc, Kyoto) with a detection limit of 2 ng/mL. Serum creatinine and total cholesterol was measured by enzymatic methods. HDL-cholesterol were determined by homogenous assays (Denka Seiken, Tokyo), and Non-HDL-cholesterol was calculated by subtracting HDL-cholesterol from total cholesterol. Body mass index (BMI) was calculated as body weight (kg) divided by squared height (m^2^).

### Statistics

Because skewed distribution was found for CRP and TRX in preliminary analyses, all continuous data were summarized as median (25th-75th percentile levels). Categorical data were given in number or percentage. Correlation was evaluated by non-parametric Spearman's rank correlation test. Difference in median levels between groups was examined by Mann-Whitney's U-test. Multiple regression models were used to evaluate independent associations, to which CRP and TRX were entered after log-transformation to fit the linear models. P-values less than 0.05 was taken to be statistically significant. All these calculations were performed with StatView 5 software (SAS Institute Inc., Cary, NC) for Windows personal computers.

## Results

### eGFR and CRP

**Figure 2:**
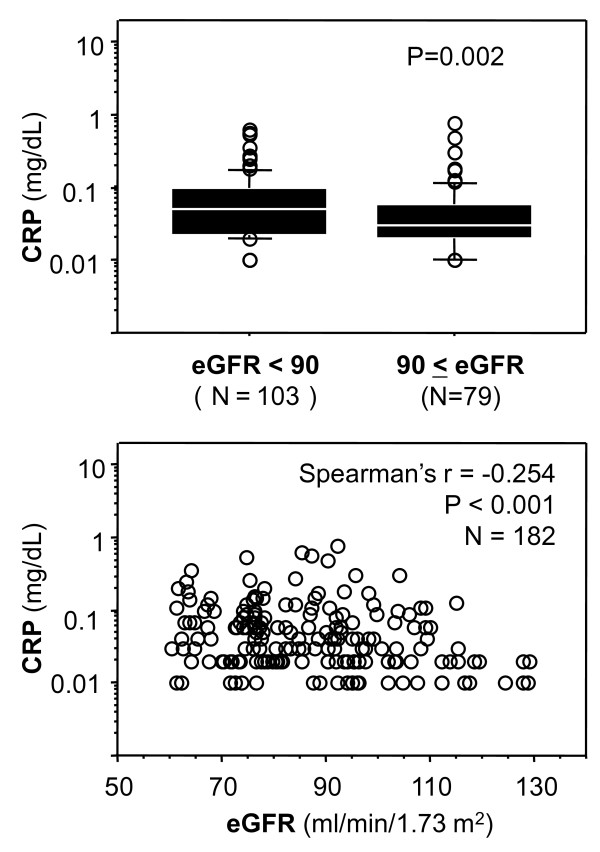
**Relationship between eGFR with CRP**. Mann-Whitney U-test was used for comparison between median levels. Correlation was evaluated by Spearman's rank correlation method. Horizontal bars indicate 10th, 25th, 50th (median), 75th, and 90th percentile levels. Abbreviations: r, correlation coefficient; eGFR, estimated glomerular filtration rate; CRP, C-reactive protein.

Figure 2 shows the relationship between eGFR and CRP. When compared between those with normal eGFR and those with mildly reduced eGFR, the median CRP level was significantly higher in the group with mildly reduced eGFR. CRP was inversely correlated with eGFR in the total subjects.

### eGFR and TRX

**Figure 3:**
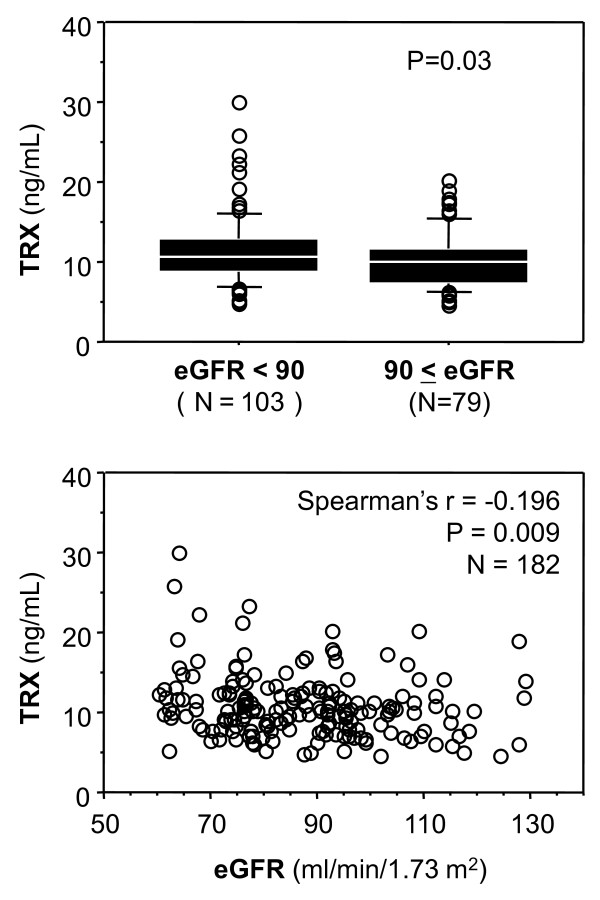
**Relationship between eGFR with TRX**. Mann-Whitney U-test was used for comparison between median levels. Correlation was evaluated by Spearman's rank correlation method. Horizontal bars indicate 10th, 25th, 50th (median), 75th, and 90th percentile levels. Abbreviations: r, correlation coefficient; eGFR, estimated glomerular filtration rate; TRX, thioredoxin.

Figure 3 shows the relationship between eGFR and TRX. The median TRX level was significantly higher in the group with reduced eGFR. TRX was inversely correlated with eGFR in the total subjects.

### Other factors correlating with CRP and TRX levels

We examined other factors that may affect the levels of CRP and TRX (Table 2). CRP was positively correlated with age, BMI, systolic BP, non-HDL-C levels, and glucose levels, whereas CRP inversely correlated with HDL-C. TRX was positively correlated with age, BMI, and systolic BP, and inversely with HDL-C. TRX showed no significant correlation with plasma glucose or non-HDL-C. CRP and TRX showed a significant positive correlation.

**Table 2:** Univariate correlation of CRP and TRX with various clinical parameters

Independent variables	Dependent variables	
	
	CRP	TRX
eGFR	-0.254***	-0.196**
Age	0.307***	0.165*
Sex	0.199**	0.298***
BMI	0.377***	0.188*
Systolic BP	0.316***	0.219**
Non-HDL-C	0.300***	0.059
HDL-C	-0.381***	-0.177*
Smoking	0.154*	0.246**
Glucose	0.239**	0.113
TRX	0.335***	---

The table gives Spearman's correlation coefficients with level of significance. Dummy variables were used for sex (1 for male, 0 for female) and smoking (1 for smokers, 0 for non-smokers). *P < 0.05, **P < 0.01, ***P < 0.001. See the footnote of Table 1 for abbreviations.

### Correlations between eGFR and other clinical variables

As shown in Table 3, eGFR was significantly associated with age, sex, BMI, systolic BP, non-HDL-C, HDL-C, and smoking status, but not with glucose level.

**Table 3:** Univariate correlation of eGFR with various clinical variables

Independent variables	Correlation coefficient
Age	-0.591***
Sex	0.269***
BMI	-0.149*
Systolic BP	-0.334***
Non-HDL-C	-0.227**
HDL-C	0.074
Smoking	0.234**
Glucose	-0.118

The table gives Spearman's correlation coefficients with level of significance. Dummy variables were used for sex (1 for male, 0 for female) and smoking (1 for smokers, 0 for non-smokers). *P < 0.05, **P < 0.01, ***P < 0.001. See the footnote of Table 1 for abbreviations.

### Independent associations of eGFR with CRP

Because CRP and TRX showed significant correlations with other clinical parameters, multiple regression models were employed to examine whether eGFR had significant associations with CRP and TRX independent of these possible confounders. As shown in Table 4, eGFR showed an inverse association with CRP in model 1 in which no adjustment was done. In model 2, the association between eGFR and CRP was again significant after adjustment for age and sex. In models 3 and 4, the association between eGFR and CRP remained significant even after further adjustment for smoking status or glucose level. However, the association between eGFR and CRP was not significant when adjusted for BMI, SBP, Non-HDL-C, HDL-C, or TRX, in addition to age and sex.

**Table 4:** Independent association of eGFR with CRP in multiple regression models.

Model	Covariables	Beta coefficients for eGFR	**R**^2^
1	unadjusted	-0.265***	0.07***
2	Age, sex	-0.171*	0.158***
3	Model 2 + Smoking	0.240**	0.171***
4	Model 2 + Glucose	-0.169*	0.168***
5	Model 2 + BMI	-0.133	0.22***
6	Model 2 + SBP	-0.160	0.179***
7	Model 2 + Non-HDL-C	-0.151	0.216***
8	Model 2 + HDL-C	-0.153	0.238***
9	Model 2 + TRX	-0.123	0.208***

The table gives beta coefficients between eGFR and CRP, and coefficients of determination (R^2^) for whole models. CRP and TRX were log-transformed to fit the linear models. Dummy variables were used for sex (1 for male, 0 for female) and smoking (1 for smokers, 0 for non-smokers).

*P < 0.05, **P < 0.01, ***P < 0.001. See the footnote of Table 1 for abbreviations.

### Independent associations of eGFR with TRX

As shown in Table 5, eGFR showed an inverse association with TRX in model 1 in which no adjustment was done. In model 2, the association between eGFR and TRX was again significant after adjustment for age and sex. In models 3 through 8, the association between eGFR and TRX remained significant even after further adjustment for BMI, SBP, Non-HDL-C, HDL-C, smoking status, or glucose. However, the association between eGFR and TRX was not significant when adjusted for CRP in addition to age and sex.

**Table 5:** Independent association of eGFR with TRX in multiple regression models.

Model	Covariables	Beta coefficient for eGFR	**R**^2^
1	unadjusted	-0.202**	0.041**
2	Age, sex	-0.196*	0.131***
3	Model 2 + Smoking	-0.188*	0.140***
4	Model 2 + Glucose	-0.188*	0.132***
5	Model 2 + BMI	-0.185*	0.136***
6	Model 2 + SBP	-0.188*	0.143***
7	Model 2 + Non-HDL-C	-0.193*	0.132***
8	Model 2 + HDL-C	-0.193*	0.133***
9	Model 2 + CRP	-0.153	0.183***

The table gives beta coefficients between eGFR and TRX, and coefficients of determination (R^2^) for whole models. CRP and TRX were log-transformed to fit the linear models. Dummy variables were used for sex (1 for male, 0 for female) and smoking (1 for smokers, 0 for non-smokers).

*P < 0.05, ***P < 0.001. See the footnote of Table 1 for abbreviations.

### Multiple regression models to simultaneously adjust for potential confounders

To further investigate the eGFR-CRP and the eGFR-TRX links, we included all potential confounders simultaneously in multiple regression models (Table 6). CRP showed significant and independent associations with systolic BP (positively), non-HDL-C (positively), and HDL-C (inversely), but not with eGFR (P = 0.16). TRX showed a significant association with male sex, and trend of association with eGFR (P = 0.06) and systolic BP (P = 0.08) at borderline significance.

**Table 6:** Multiple regression analyses simultaneously including all potential confounding variables

Independent variables	Dependent variables	
	
	CRP	TRX
**eGFR**	-0.114	-0.168
**Age**	0.080	0.046
**Sex**	0.040	0.208*
**Smoking**	0.094	0.123
**Glucose**	0.007	-0.094
**BMI**	0.125	0.085
**Systolic BP**	0.164*	0.145*
**Non-HDL-C**	0.158*	-0.025
**HDL-C**	-0.200*	-0.017

**R**^2^	0.305***	0.164***

The table gives beta coefficients between eGFR and CRP and between eGFR and TRX, and coefficients of determination (R^2^) for whole models. CRP and TRX were log-transformed to fit the linear models. Dummy variables were used for sex (1 for male, 0 for female) and smoking (1 for smokers, 0 for non-smokers). See the footnote of Table 1 for abbreviations.

*P < 0.05, ***P < 0.001.

## Discussion

The aim of this study was to compare the levels of CRP and TRX between subjects with normal and mildly reduced renal function. When the subjects were divided into two groups by eGFR, both CRP and TRX were higher in the subjects with mildly reduced eGFR. Also, eGFR showed significant inverse correlations with CRP and TRX in the total subjects. The inverse associations of eGFR with CRP and TRX remained significant after adjustment for age and sex. When further adjustment was done for 6 additional possible confounders, the inverse associations of eGFR with CRP and TRX became less significant. In such models, CRP was independently associated with systolic BP, non-HDL-C, and HDL-C levels. Also, TRX was associated with sex significantly, and with eGFR and systolic BP at border significance. These results suggest that the increased levels of CRP and TRX in subjects with mildly reduced eGFR were mediated, at least partly, by alterations in blood pressure and lipid levels in mildly decreased kidney function.

Previous studies reported that patients with advanced renal failure have increased levels of CRP and biomarkers for oxidative stress including TBARS[14,15], phosphatidylcholine hydroperoxide[14], F_2_-isoprostane[31], and AOPP[15]. However, there are only a few studies that examined oxidative stress in those with mild reduction in renal function. According to Witko-Sarsat et al[33], plasma AOPP levels were increased early in the course of CKD, and further increased in more advanced renal failure. Fortuno et al[34] showed that patients with stage 1-2 CKD had an increase in phagocytic NADPH oxidase-dependent superoxide production in as compared with healthy control subjects. Regarding antioxidant defense in early CKD, Yilmaz et al[35] reported that erythrocytes from patients with stage 1-2 CKD had lower activities of SOD and glutathione peroxidase than healthy controls. These previous studies suggested the increased oxidative products and impaired antioxidant defense even in early stages of CKD. However, no study examined possible changes in the biomarkers for inflammation and oxidative stress among subjects with mild reduction in eGFR as compared to those with normal eGFR. In addition, these previous studies did not made correction for possible confounding variables, due presumably to small number of subjects. The present study compared CRP and TRX levels between those with normal and mildly reduced eGFR, and showed that mild reduction in eGFR was associated with increased levels of CRP and TRX in dependent of age and sex using multivariate analyses in 182 subjects. These data provide further evidence supporting the notion that inflammation and oxidative stress are increased in a very early course of renal function loss.

In this study, eGFR, CRP, and TRX were correlated with each other. Importantly, the association of eGFR and CRP was not significant after adjustment for TRX in addition to age and sex. Similarly, the association of eGFR and TRX was not significant after adjustment for CRP in addition to age and sex. These results suggest the close association among inflammation, oxidative stress, and renal function. Subjects with early stages of CKD have increased NADPH oxidase activity[34] and compromised antioxidant defense mechanisms[35]. These data may indicate that impaired renal function is the cause of increased oxidative stress. Conversely, since increased oxidative stress causes organ damage, the increased oxidative stress due to reduced GFR could, in turn, further impair kidney function [38]. In addition, inflammation may increase oxidative stress[15,31,39,40], and also promote loss of kidney function [41]. Furthermore, renal insufficiency results in sustained inflammation, since some inflammatory cytokines are excreted through kidneys[42]. Thus, these studies suggest the complex inter-relationship among decreased renal function, increased oxidative stress, and inflammation.

Furthermore, the present study indicates possible contributions of blood pressure and plasma lipids to the eGFR-CRP link and the eGFR-TRX link. In the fully-adjusted models, eGFR was not significantly associated with either CRP or TRX, whereas CRP was significantly associated with systolic BP, HDL-C, and non-HDL-C levels. TRX was associated with systolic BP at borderline significance. Since both blood pressure and plasma lipids are adversely affected by impaired kidney function, and these are well known risk factors for atherosclerosis, we speculate that the increased levels of CRP and TRX in subjects with mildly reduced eGFR were mediated, at least partly, by alterations in blood pressure, plasma lipids and presumably arterial wall in such subjects.

We interpret the increased TRX levels associated with mildly reduced eGFR to indicate that oxidative stress is increased in those with mildly reduced renal function. However, we cannot exclude other possibilities. The increased TRX may be due simply to retention of TRX in decreased renal function. So far, it is unknown to what extent glomerular filtration is involved in the elimination of TRX from the circulation. According to Kasuno et al[18], TRX is detectable in urine of healthy individuals, and urinary TRX is increased in some kidney diseases. They also demonstrated the translocation of TRX from renal tubular cells into urinary lumen in response to ischemia/reperfusion in mice. Thus, urinary TRX may represent 'leak' of TRX from damaged kidney cells rather than glomerular filtration of the protein.

This study has several limitations. First, GFR was not directly determined but estimated by the formula that was developed for and validated in Japanese subjects. Therefore, direct GFR determination would be needed to obtain more solid conclusion. Second, because of the cross-sectional design of this study, the associations between parameters did not necessarily indicate causality. Prospective studies will be required for this purpose. Third, the subjects of this study do not represent the general population although we recruited them from the participants of a health check-up program. They included more women than men, and did not include those taking medications for the three common diseases.

## Conclusions

In conclusion, subjects with mildly decreased eGFR showed increased levels of biomarkers for inflammation and oxidative stress. This finding is of clinical importance, since CKD is recognized as a very high-risk population for CVD. Further studies are necessary whether the observed deviations in the biomarkers of inflammation and oxidative stress are predictive of occurrence of CVD.

## Competing interests

The authors declare that they have no competing interests.

## Authors' contributions

ST acquired data, analyzed, interpreted data, and drafted the manuscript. TS designed the study, analyzed, interpreted data, and drafted the manuscript. NS and RK acquired data. ME, HK, EI, MI, and YN interpreted data and drafted the manuscript. TS had full access to all the study data and assume responsibility for the integrity of the data and the accuracy of the analysis. All authors read and approved the final manuscript.

## Pre-publication history

The pre-publication history for this paper can be accessed here:

http://www.biomedcentral.com/1471-2369/11/7/prepub
